# Zr-Y-Nb-REE mineralization associated with microgranite and basic dykes at EL Sela shear zone, South Eastern Desert, Egypt

**DOI:** 10.1186/2193-1801-3-573

**Published:** 2014-10-01

**Authors:** Hassan Abd El-Razek Aly Shahin

**Affiliations:** Nuclear Materials Authority, P. O. Box 530, El-Maadi, Cairo, Egypt

**Keywords:** Zr-Y-Nb-REE mineralization, Microgranite dyke, Basic dyke

## Abstract

El Sela shear zone occurs in the younger granite rock of Gabal El Sela area, south Eastern Desert, Egypt near the Sudan Frontier. It comprises lines–arranged intrusions trending ENE-WSW and extend for about 1.5 km in length and reach up to 40 meters in width. These lines–arranged intrusions include multi-phase quartz veins, altered microgranite and altered basic dykes. These dykes hosting or acting as a source for uranium, rare metals (Zr, Y, Nb and Ga) and light rare earths (La, Ce, Sm and Nd) mineralizations. They show highly alteration, uranium enrichment and a strong enrichment in some rare metals and light rare earths contents (Zr = 644, Y = 133, Nb = 136, Ga =184, La = 50.19, Ce = 105.47, Sm = 24.81, Nd = 78.91 ppm and and ∑ LREEs = 259.38.). The chondrite normalised rare earth elements trends indicate strongly fractionated rare earth elements pattern with significant enriched of LREE according to HREE in both altered microgranite and altered basic dykes. Field radiometric measurements of the studied altered microgranite dyke revealed that eU reach up to 359 ppm with an average 78 ppm, while in the altered basic dyke reach up to 1625 ppm with an average 144 ppm.

## Introduction

**Figure 1:**
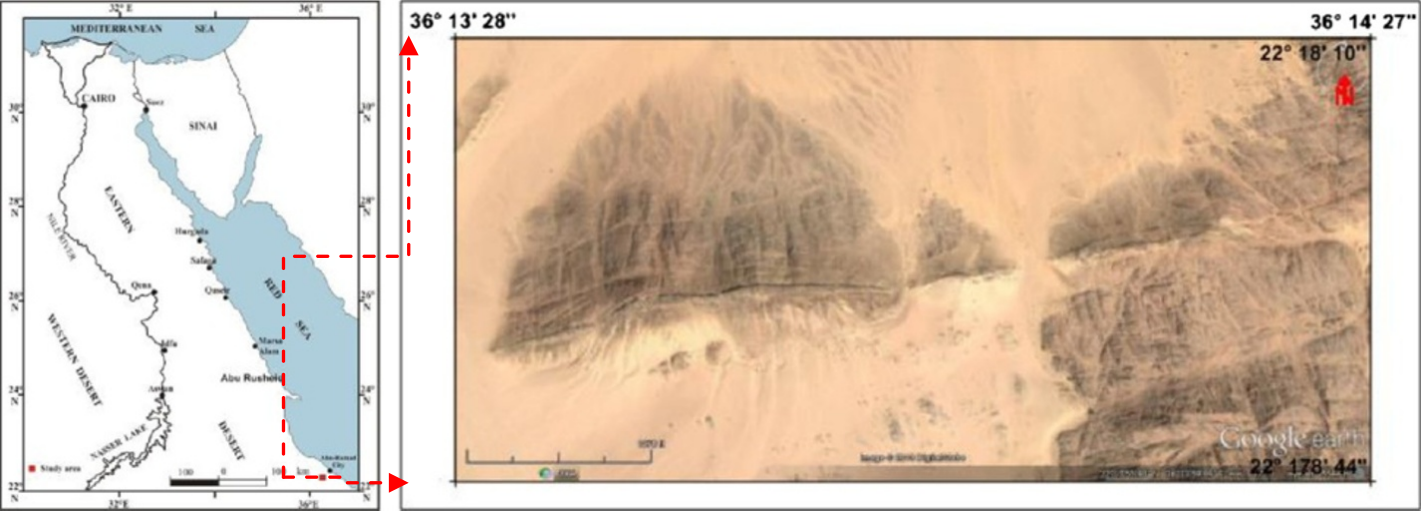
**Location map and Google image for the studied area.**

El Sela shear zone is located in Gabal El Sela area in the southern extremity of the Eastern Desert of Egypt near the Sudan Frontier and occupies the southern half of Elba topographic sheet (NF-37 I). It lies at a distance of about 22 km SW of Abu-Ramad city (Figure 1). It is bound by Latitudes 22° 17' 50" – 22° 18' 6" N and Longitudes 36° 13' 36" - 36° 14' 22" E. The geology, mineralogy, geochemistry and radiometry of El Sela area was studied by several authors, e.g., Basta and Saleeb (1971), Hussein et al. (1973), Hussein (1977), El-Gaby et al. (1988), Nasr and Youssef (1995), Assaf et al. (1999), Abdel-Meguid et al. (2003), Khalaf (2005), Gaafar (2005), Ibrahim et al. (2005), Abd Elaal (2006), Gaafar et al. (2006), Abu Donia (2006), Ibrahim et al. (2007), El Afandy et al. (2008), Abd El-Naby and Dawood (2008), Ali (2011), Ali and Lentz (2011), Shahin (2011), Bayoumi (2011), Gamil et al. (2012), Aly (2013) and Abdel Gawad et al. (2014).

The most important studies are Gaafar et al. (2006), who studied the gamma ray spectrometry of a promising vein type uranium mineralization associated with El Sela granite, and concluded that the detailed ground gamma ray spectrometric survey on Sela shear zone clearly reflects the outline of this zone, where the sheared lamprophyre dyke of this zone show sharp increase of the eU contents and Aly and Lentz (2011), they studied the mineralogy, geochemistry and age dating of shear zone-hosted Nb-Ta-, Zr-Hf-, Th and U-bearing granitic rocks in the Ghadir and El-Sella areas, South Eastern Desert, Egypt. They concluded that the rare metal minerals of mineralized altered granites within El-Sella shear zones are columbite-tantalite minerals as ferrocolumbite, pyrochlore, and fergusonite, Th-minerals (cheralite, uranothorite, and huttonite monazite), Hf-zircon, monazite and xenotime.

The present paper summarizes the geology, petrology, geochemistry and mineralization of the altered microgranite and altered basic dykes cutting in El Eela shear zone, south Eastern Desert, Egypt.

### Field studies

#### 1. Geologic setting

**Figure 2:**
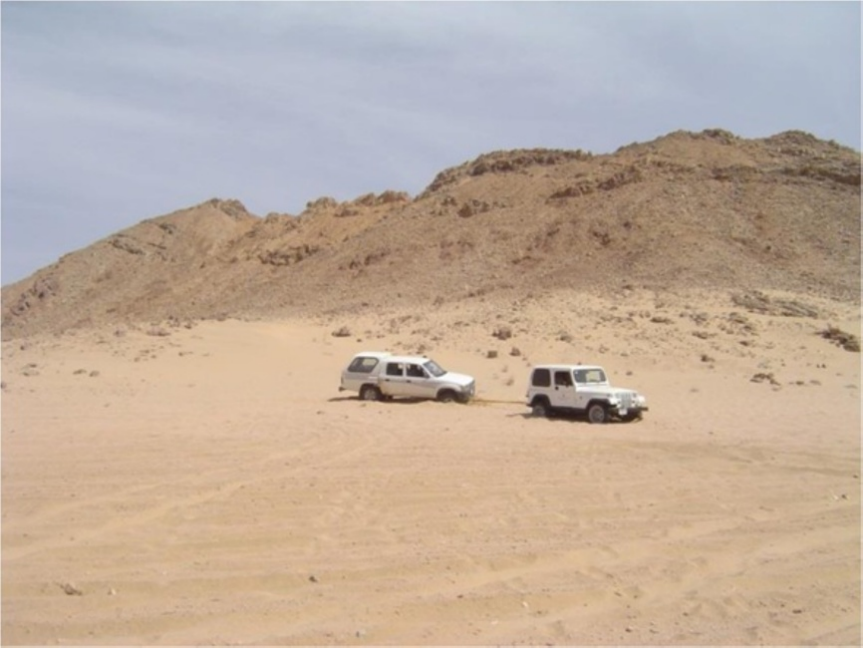
**Lines–arranged intrusions include multi-phase quartz veins, altered microgranite and altered basic dykes at El Sela Shear zone.** Photo looking E.

**Figure 3:**
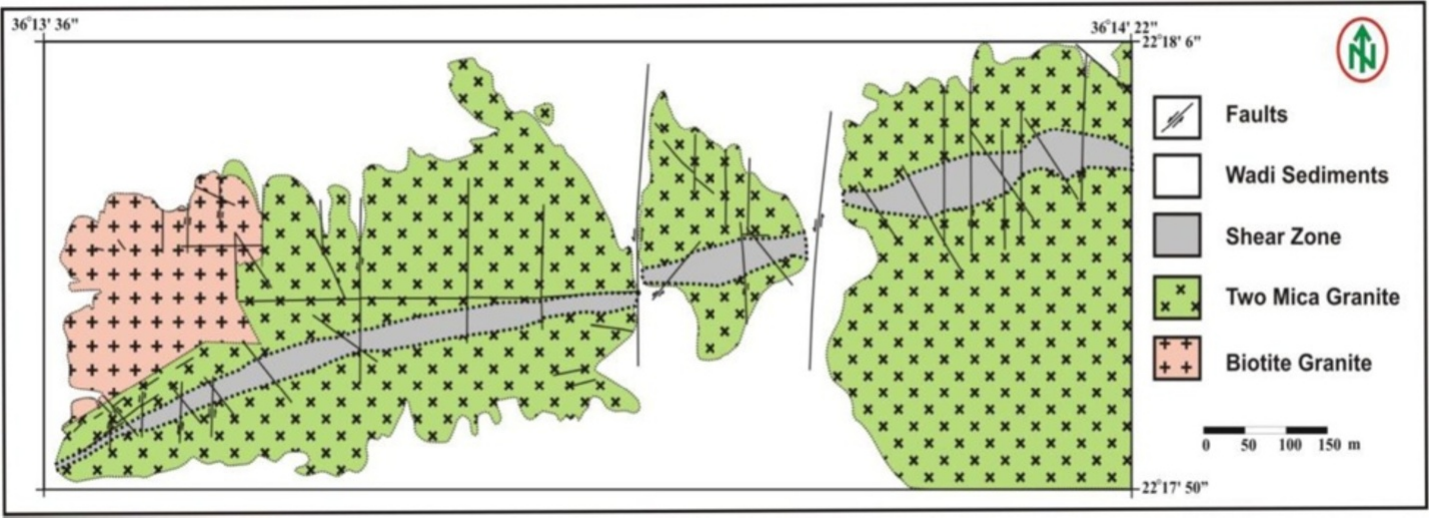
**Geologic map of the El Sela shear zone, south Eastern Desert, Egypt, after Abdel-Meguid et al.** (2003)**.**

**Figure 4:**
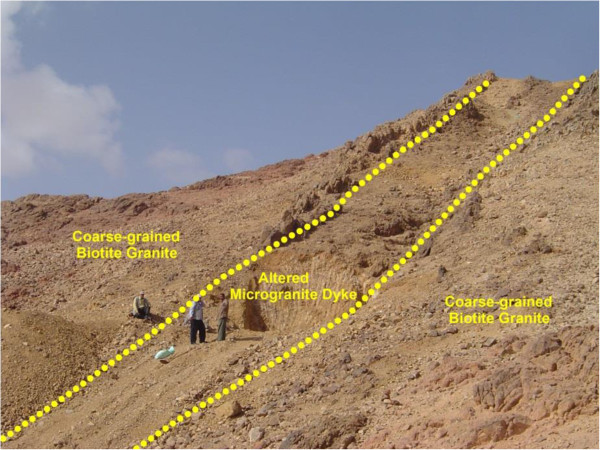
**Photograph showing microgranite dyke cuts in coarse-grained biotite granite.** Photo looking W.

**Figure 5:**
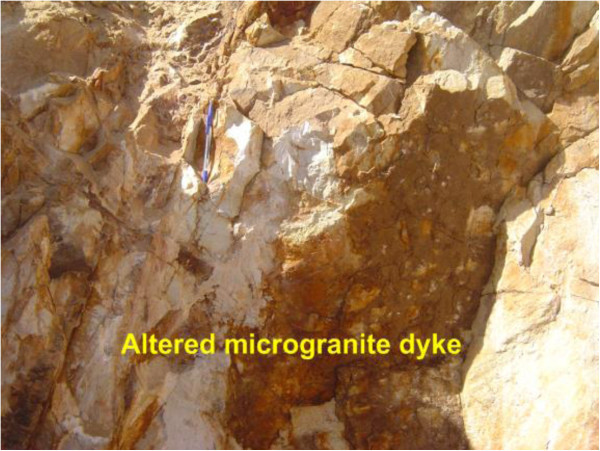
**Close view showing altered microgranite dyke.** Photo looking W.

El Sela shear zone comprising lines–arranged intrusions trending ENE-WSW and extends for about 1.5 km in length and reach up to 20 meters in width. These lines–arranged intrusions include multi-phase quartz veins, altered microgranite and altered basic dykes (Figure 2). A detailed geologic study is carried out on the biotite ± muscovite granite of El Sela shear zone (Figure 3). This granite is highly weathered, cavernous and exposed as low to moderate separately hills, coarse-grained, pink to pinkish gray in color, mainly composed of K-feldspar, quartz, plagioclase, biotite and rare muscovite. It is characterized by the presence of iron and manganese oxides filling joints and fractures indicating the enrichment of these mineralizations. This granite is also enriched with altered pyrite and occasionally leach out leaving cubic vugs and patches of dark red color of hematitization. This granite is intruded by microgranite and basic dykes. The microgranite occurs as dyke and sheets, mainly injected along the ENE-WSW direction (Figures 4 and 5). This dyke is whitish buff to buff, leucocratic, fine grained, massive, equigranular texture and composed mainly from quartz, plagioclase K-feldspare and biotite. It is very rich in pyrite and characterized by the presence of extremely abundant manganese oxides filling the joints and fractures. Thickness of this dyke varies from 30 cm to 5 meters and extends for more than 1500 meters, appear and disappear along the ENE-WSW direction. Locally; it is completely altered to pale pinkish brown color enriched by box work vugs of the dissoluted pyrite. Sometimes, it is characterized by porphyritic texture with prominent phenocrysts of plagioclase and quartz minerals. Field relations indicate that this microgranite dyke is dissected by basic dyke.

**Figure 6:**
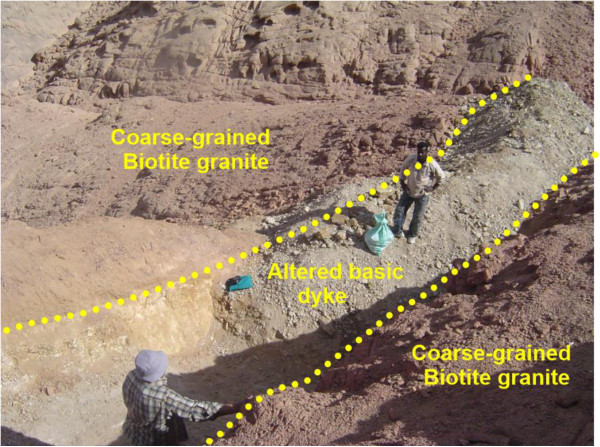
**Photograph showing altered basic dyke cuts in coarse-grained biotite granite.** Photo looking SW.

**Figure 7:**
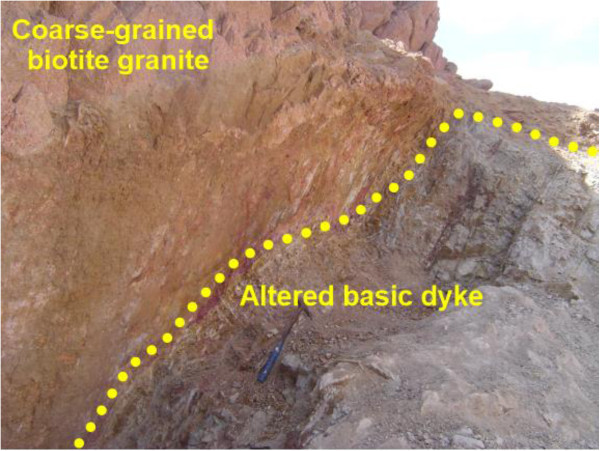
**Close view showing altered basic dyke enriched by iron oxides.** Photo looking S.

The basic dyke follow the main fault planes with subvertical to steep dipping to the south. It extends along the ENE-WSW shear zone for more than 1.5 km long, appear and disappear in some areas with width varies from 1 meter to 5 meters. It is dark gray to grayish green color, fine grained, mostly altered, enriched by iron oxides and composed essentially of plagioclase, amphibole, chlorite, epidote and little quartz. Sometimes, it is characterized by porphyritic textures with prominent phenocrysts of both orthoclase and quartz minerals. They have higher uranium content many times greater than the hosting granite. The uranium contents reach up to 1625 ppm eU with relatively high thorium content. Field relations indicate that this dyke introuded both coarse-grained biotite granite and microgranite dyke with sharp contact (Figures 6 and 7).

#### 2. Structural geology and mineralization

**Figure 8:**
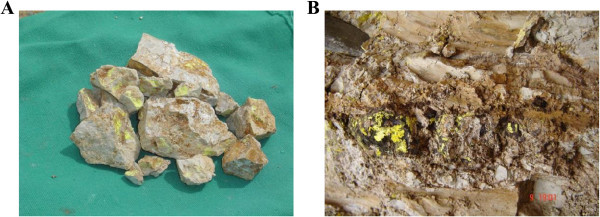
**Uranium mineralization, A. Uranophane associated with altered microgranite dyke, B. Uranophane associated with altered basic dyke.**

**Figure 9:**
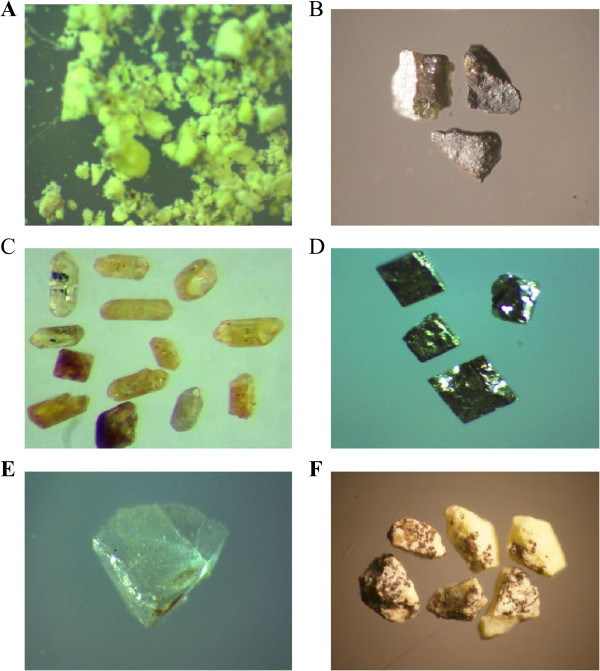
**Minerals associated with altered microgranite dyke, A. Uranophane mineral (PPL. X20), B. Columbite mineral (PPL. X10), C. Zircon mineral (PPL. X10), D. pyrite mineral (PPL. X40), E. Colorless fluorite mineral (PPL. X20), F. Uranophane associated with fergusonite mineral (PPL. X10).**

**Figure 10:**
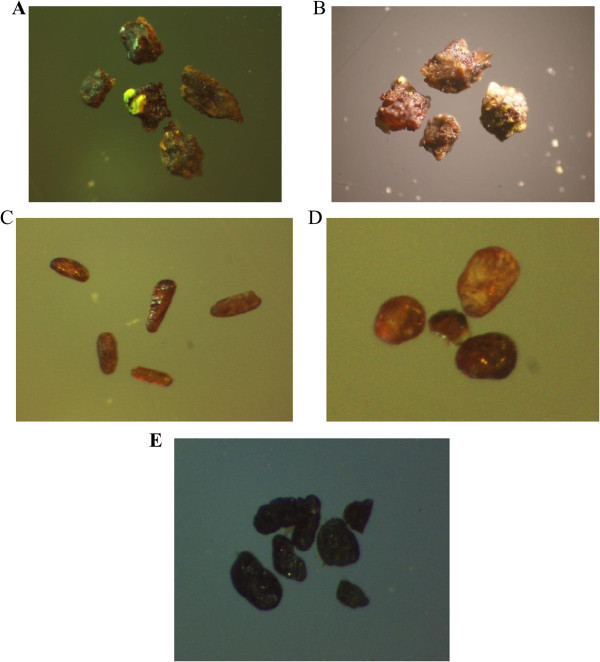
**Minerals associated with altered basic dyke, A. Samarskite mineral associated with uranophane (PPL. X10), B. Fergusonite mineral associated with uranophane (PPL. X10), C. Rutile mineral (PPL. X10), D. Sphene mineral (PPL. X10), E. Rutile-columbite mineral (PPL. X20).**

The area is intersected by several fault sets and crosscut by different microgranite, basic and lamprophyre dykes in various thickness and directions. The main tectonic trends affect the studied granite are E-W to ENE-WSW and N-S fault sets with contemporaneous different injections. The emplacement of these injections along the fault zones were associated with high potential fluids whic are indicated by the alteration halos. Hematitization and kaolinitization are the main alteration processes in the area. El Sela shear zone (ENE-WSW) trend is an important structural feature in Gabal El Sela area south Eastern Desert, Egypt. It is spatially and genetically associated with all the major uranium deposits (Figure 8A and B). El Sela shear zone was involved in a long and complex history of deformation, alterations and mineralizations. Repeated reactivation along major fault trending ENE-WSW over a protracted period is revealed through a range of fault rocks, spanning from mylonites to breccias and veins. The faulted rocks are filled by multi-phase quartz veins, altered microgranite and altered basic dykes. Each tectonic event is associated with a period of uranium mineralization, with the major mineralization hosted by brecciated altered microgranite dyke and altered basic dyke.The tectonic-magmatic-hydrothermal evolution of the study area is suggested in several sequences of hydrothermal fluids. The first one associated with the altered microgranite dyke who indicates the existence of hydrothermal/mineralizing event (uranophane, columbite, zircon, colorless fluorite and pyrite) (Figure 9). The second one also associated with altered microgranite dyke and suggests the deposition of fergusonite mineral on uranium mineralization (Figure 9F). The third one associated with the altered basic dyke who indicates the existence of hydrothermal/mineralizing event (uranophane, samarskite, fergusonite, rutile, rutile-columbite, sphene, violt fluorite, pyrite and ilmenite) (Figure 10).

#### 3. Radiometric investigation

**Figure 11:**
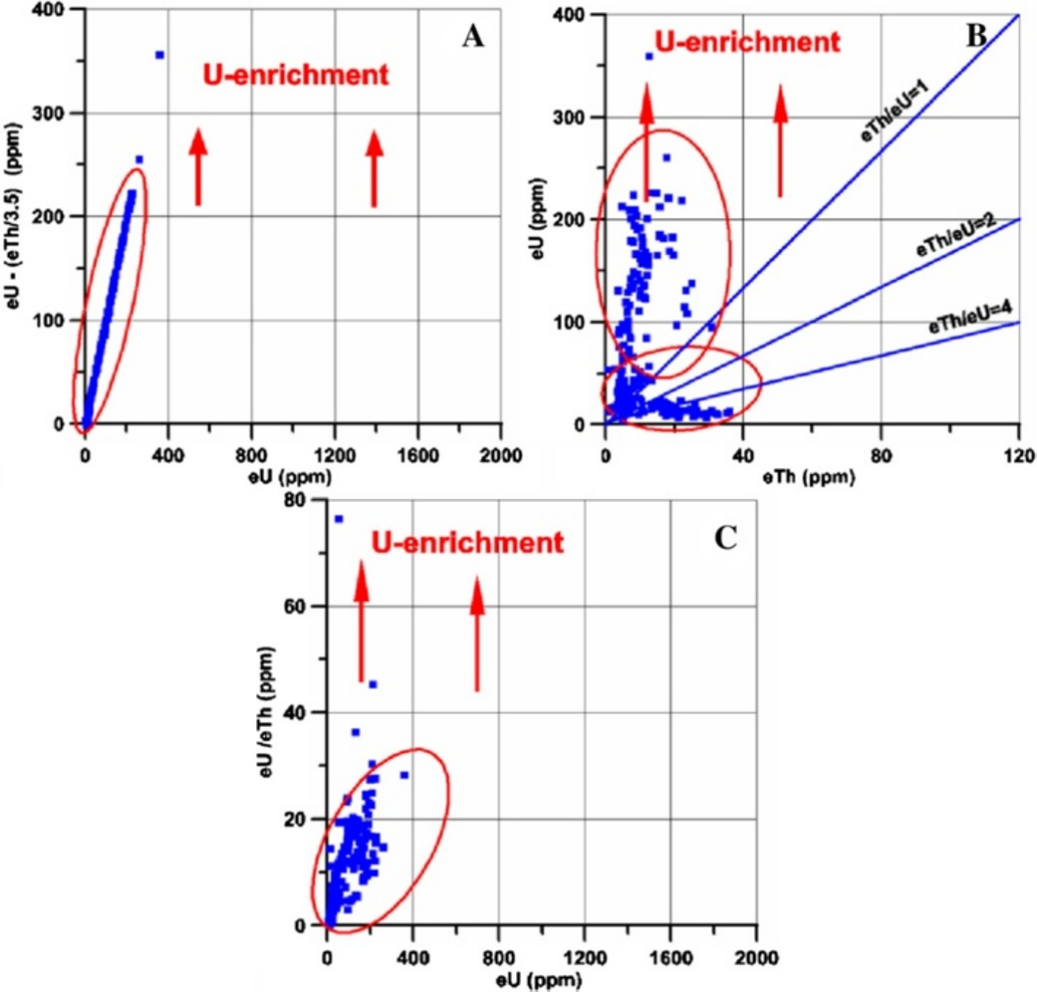
**Radioactive elements plots for ground gamma-ray spectrometry measurements in the altered microgranite dyke, A. Mobility, B. eTh versus eU, C. eU versus eU/eTh.**

**Figure 12:**
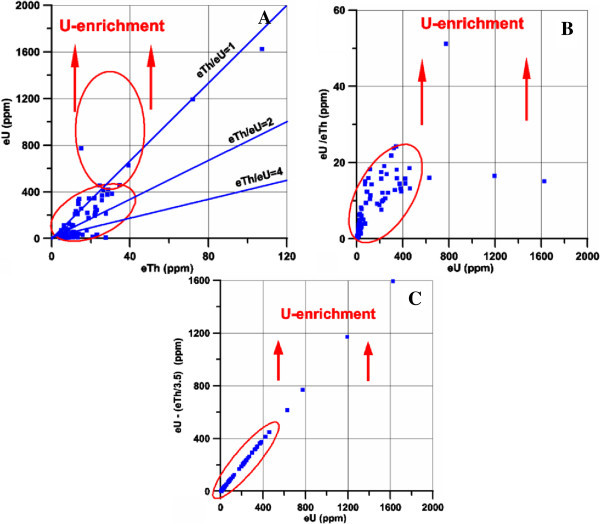
**Radioactive elements plots for ground gamma-ray spectrometry measurements in the altered basic dyke, A. Mobility, B. eTh versus eU, C. eU versus eU/eTh.**

Detailed field radiometric measurements using a portable four channel, gamma-ray spectrometer Model GR-230 was carried out along the altered microgranite and alteref basic dykes at El Sela shear zone. The radiometric measurements revaled that the eU reaching up to 359 ppm in the altered microgranite dyke, and reaching up to 1625 ppm in the altered basic dyke. The statistical treatment of spectrometric data was expressed on binary diagrams of eTh versus eU, eU versus eU/eTh and eU versus eU- eTh/3.5 (Figures 11 and 12). These figures indicate that both the altered microgranite and the altered basic dykes hosted high uranium content.

### Petrography

**Figure 13:**
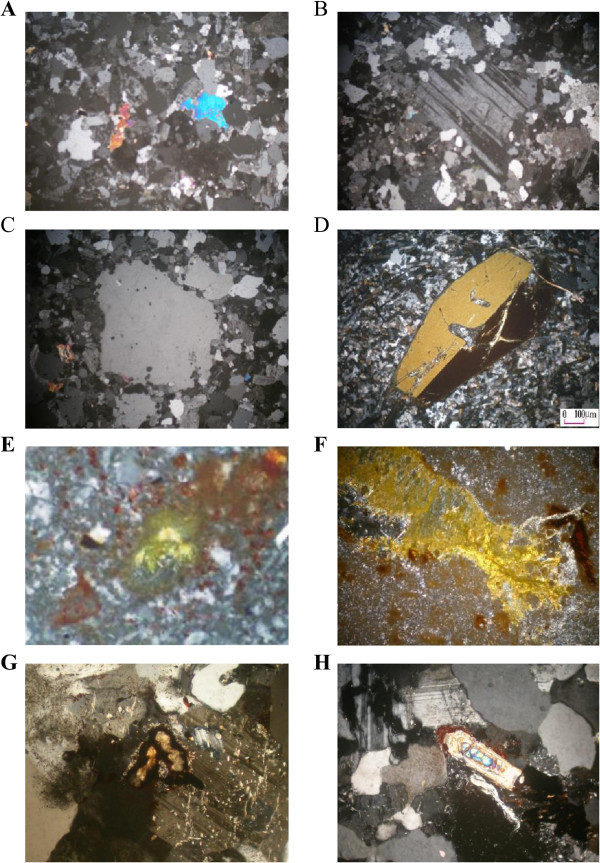
**Microscopic investigation of the studied altered microgranite and altered basic dykes A. Equigranular texture of microgranite dyke (C.N. X40), B. Plagioclase phenocrysts embedded in microcrystalline groundmass in microgranite dyke (C.N. X40), C. Quartz phenocrysts embedded in microcrystalline groundmass in microgranite dyke (C.N. X40), D. Orthoclase phenocrysts embedded in microcrystalline groundmass in basic dyke (C.N. X40), E. Uranothorite associated with iron oxide in microgranite dyke (C.N. X 40), F. Uranophane associated with groundmass of kaoline and iron oxide in basic dyke (C.N. X40), G. Uranothorite associated with iron oxide in microgranite dyke (C.N. X40),H. Metamict zircon associated with perthite and albite plagioclase in microgranite dyke (C.N. X40).**

Petrographically, the microgranite dyke display strong alteration. It is whitish buff to buff, leucocratic, fine grained, massive, equigranular texture and composed essentially of quartz, microcline, perthite, plagioclase, biotite and muscovite (Figure 13A). Quartz forms anhedral to irregular crystals (0.1-0.4 mm), microcline-perthite occurs as anhedral to subhedral crystals filling the interstices between the plagioclase and quartz (0.2-0.5 mm), plagioclase forms short prismatic crystals (0.2-0.6 mm), biotite often chloritized, has dark pleochroic colors (0.1-0.3 mm). Muscovite occurs as minute crystals enclosed in plagioclase and filling the interstices between plagioclase and quartz (0.1- 0.4 mm). Sometimes it is characterized by porphyritic textures with prominent phenocrysts of both plagioclase and quartz minerals (Figures 13B and C).

The basic dyke shows intense alteration to be completely altered to kaoline, silica and hematite without any relics of original rock-forming minerals and texture. Under the microscope, it is composed of kaoline, quartz, muscovite, iron oxides. Kaolinite occurs as alteration product of calcic-plagioclase. Quartz occurs as cryptocrystalline silica veinlets filling fractures in kaoline.Muscovite occurs due to alteration product of kaoline by acidic solution rich in K+. Iron oxide occurs as fine grained aggregates. Occasionally it is characterized by the presence of orthoclase phenocrysts (Figure 13D).Uranophane, uranothorite and zircon are the most important accessory minerals in these dykes (Figures 13E, F, G and H). Uranophane occurs as anhedral crystals of yellow color associated with iron oxide. Uranothorite occurs as subhedral crystals of orange, yellowish-orange color associated with iron oxides. Zircon occurs as metamict zoned zircon of prismatic shape with bipyramidal terminations intergrown, mostly is subhedral to euhedral crystals associated with quartz, perthite and albite plagioclase.

## Material and methods

Thirty-seven samples from the best exposures of the microgranite and basic dykes were collected for this study. From these, 10 samples were selected for thin sections to study the mineral constituents of these dykes. Fifteen representative samples of microgranite dyke and twelve representative samples of basic dyke were selected and chemically analyzed for their major oxides trace elements and rare earth contents. The trace elements were analyzed using X-ray fluorescence analyzer (XRF) and the rare earth elements were analyzed using inductively plasma (IP) and Inductively Coupled Plasma Mass Spectrometry (ICP-MS) techniques. The analyses were performed in the Nuclear Materials Authority Laboratory, Cairo, Egypt. Data of the major oxides, trace and rare earth elements of the studied dykes are listed in Tables 1, 2, 3 and 4.

**Table 1:** **Trace elements (ppm) petrochemical data for the altered microgranite dyke at El Sela shear zone**

S.No.	Cr	Ni	Cu	Zn	Zr	Rb	Y	Ba	Pb	Sr	Ga	V	Nb
**ES-213/1**	48	18	44	264	438	291	94	1502	23	2393	20	475	95
**ES-203**	59	21	40	61	161	242	11	2003	71	254	38	93	8
**ES-202**	70	12	35	42	56	272	4	395	89	80	48	17	3
**ES-201**	59	21	41	60	46	199	4	370	161	66	83	33	3
**ES-200**	69	23	38	51	135	239	9	1491	37	209	20	59	7
**ES-38**	147	131	70	43	85	5	6	3764	9	137	12	148	4
**ES-35/1**	98	23	40	80	131	216	9	3941	108	195	51	174	7
**ES-39/2**	96	28	34	57	117	152	8	3116	111	186	52	132	6
**T-12**	53	12	34	121	38	209	3	236	142	50	60	12	2
**T-8**	96	24	33	38	95	179	7	2705	63	141	32	111	5
**T-232/1**	37	11	40	211	370	-	25	4546	412	577	184	120	19
**ES-202/1**	58	10	36	24	30	163	2	226	36	45	22	10	2
**ES-40**	95	26	38	62	117	203	8	2983	65	186	35	119	6
**T-9**	102	23	53	75	132	59	10	3304	166	185	100	121	7
**T-7/2**	114	34	45	174	128	134	9	4794	44	193	21	183	7

**Table 2:** **Trace elements (ppm) petrochemical data for the altered basic dyke at El Sela shear zone**

S.No.	Cr	Ni	Cu	Zn	Zr	Rb	Y	Ba	Pb	Sr	Ga	V	Nb
**BC**	218	88	53	109	160	425	35	790	56	860	49	370	35
**ES-232**	51	16	40	253	486	367	103	1483	25	2684	26	462	105
**ES-213**	68	19	40	175	248	343	53	1095	56	1337	47	368	53
**ES-209**	31	13	42	392	627	134	133	2015	87	3420	68	605	136
**ES-41**	31	11	35	133	644	-	45	8646	309	964	118	215	34
**ES-39/1**	237	89	49	128	179	479	38	883	19	993	22	375	39
**ES-37/1**	193	60	62	431	137	188	29	827	43	764	40	353	29
**ES-36/1**	177	59	45	105	172	277	36	773	28	980	24	330	37
**T-9/1**	30	12	39	138	579	214	115	1346	29	3345	24	407	123
**T-8/1**	105	49	44	90	187	368	13	4105	23	296	22	146	10
**T-6**	45	8	38	29	69	231	5	308	27	102	16	12	4
ES-6/3	118	44	42	85	140	172	10	3873	61	216	29	162	7

**Table 3:** **Rare earth elements (ppm) petrochemical data for the altered microgranite dyke at El Sela shear zone**

S.No.	T8	T9	T12	ES 35/1	ES 40
**La**	15.20	2.30	4.86	4.66	50.19
**Ce**	34.50	5.06	12.20	10.42	105.47
**Pr**	4.35	0.69	1.55	1.55	16.49
**Nd**	17.28	2.78	7.75	8.94	78.91
**Sm**	5.15	1.05	1.98	2.19	24.81
**Eu**	0.16	0.14	0.04	0.51	0.75
**Gd**	4.5	0.98	2.10	2.80	9.86
**Tb**	0.95	0.16	0.35	0.60	3.20
**Dy**	4.6	0.95	1.66	3.88	21.00
**Ho**	1.22	0.24	0.52	0.85	4.39
**Er**	3.50	0.75	1.47	2.20	14.20
**Tm**	0.40	0.13	0.25	0.32	1.57
**Yb**	1.69	0.69	1.52	2.11	5.86
**Lu**	0.15	0.10	0.16	0.27	0.54

**Table 4:** **Rare earth elements (ppm) petrochemical data for the altered basic dyke at El Sela shear zone**

S.No.	ES6/3	ES37/1	BC	ES39/1
**La**	14	-	5.4	-
**Ce**	83.4	18.8	47.4	33.7
**Pr**	-	-	-	-
**Nd**	58.2	62	64.8	67.8
**Sm**	-	-	-	-
**Eu**	3.6	7.2	5.8	6.2
**Gd**	9.5	14.3	12.1	13
**Tb**	1.3	2.2	1.8	2
**Dy**	8.4	6.4	8.2	7.7
**Ho**	1	1.3	1.1	1.3
**Er**	-	-	-	-
**Tm**	0.4	0.3	0.41	0.42
**Yb**	-	-	-	-
**Lu**	0.2	0.3	0.32	0.35

- Not determined.

## Results

### Trace elements

**Figure 14:**
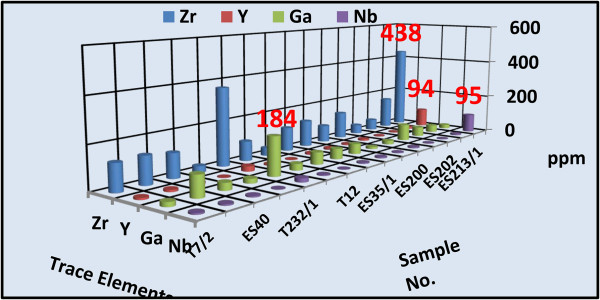
**Histogram shows the trace elements content of the altered microgranite dyke.**

**Figure 15:**
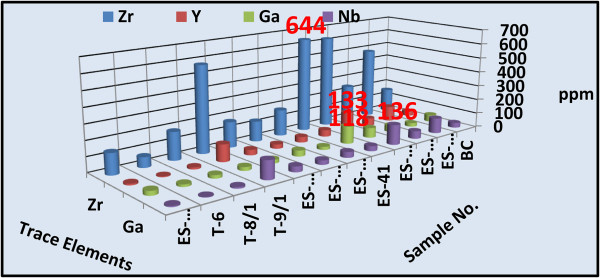
**Histogram shows the trace elements content of the altered basic dyke.**

Concentrations of a wide variety of trace elements are shown in Tables 1 and 2. These tables show a strong enrichment in some rare metal contents in both altered microgranite dyke (Zr = 438, Y = 94, Nb = 95, Ga =184 ppm) and altered basic dyke (Zr = 644, Y = 133, Nb = 136, Ga =118 ppm) respectively. Figures 14 and 15 histograms show the highest trace elements values for altered microgranite and altered basic dykes.

The higher content in some rare metal and rare earth mineralizations of the altered basic dyke relative to the altered microgranite dyke due to several reactivation of the shear zone (ENE-WSW) with normal fault movement accompanied with hydrothermal fluids lead to the intense alteration of basic dyke with completely altered to clayey materials rich with secondary uranium and some rare metal and rare earths mineralization without any relics of the primary mineralogy. These clayey materials acting as trapped phase and carrier for some rare metal and rare earth mineralizations enriched this dyke. So, these hydrothermal fluids are potentially important in the dissolution, transportation and precipitation of these elements in this dyke.

### Rare earth elements (REEs)

In most igneous systems, Zr, Nb, Y and REE are immobile during fluid-rock interaction, and for this reason are widely used as petrogenetic indicators (Pupin (1980); Hoskin and Schaltegger (2003); Schaltegger et al., (2005)). However, there is increasing evidence in our study that these elements can be transported by hydrothermal fluids under the restrictive sets of conditions that commonly prevail in alkaline igneous settings. Our study has provided evidence of metamict processes, which resulted in alteration of the cores of zircon crystals and mobilization of Y and HREE from these crystals to form minerals such as fergusonite-(Y) and Samarskite-(Y). Similar alteration has been documented by Anderson et al. (2008) for zircon from the Georgeville granite, Nova Scotia. The LREE form considerably stronger complexes with fluoride than the HREE and are therefore more easily mobilized. The REE also form relatively strong complexes with chloride and as for fluoride, the strongest complexes are with the LREE, Sheard (2010). Therefore, in our study the altered microgranite and altered basic dykes are riched in fluoride and chloride, so they enriched by LREE rather HREE.

**Figure 16:**
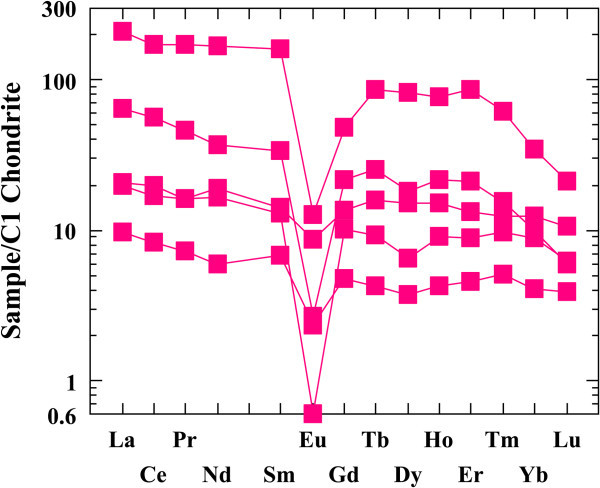
**Chonderite normalized pattern for the studied altered microgranite dyke.**

**Figure 17:**
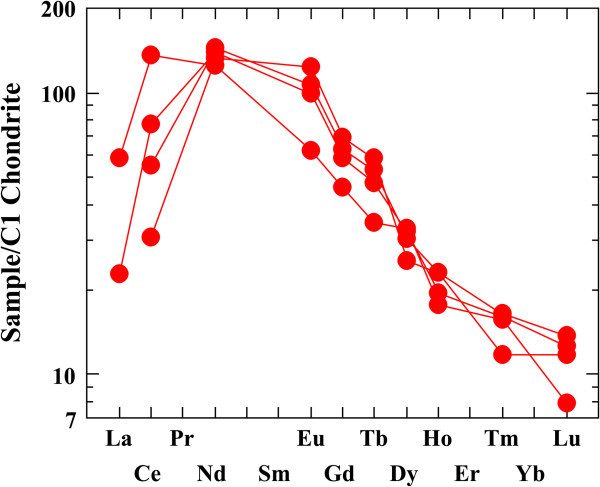
**Chonderite normalized pattern for the studied altered basic dyke.**

**Figure 18:**
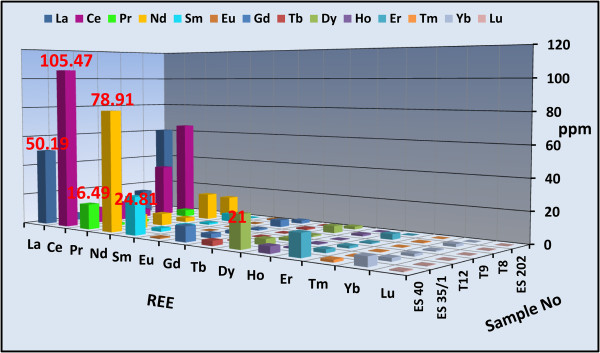
**Histogram shows the rare earth elements content in the altered microgranite dyke.**

**Figure 19:**
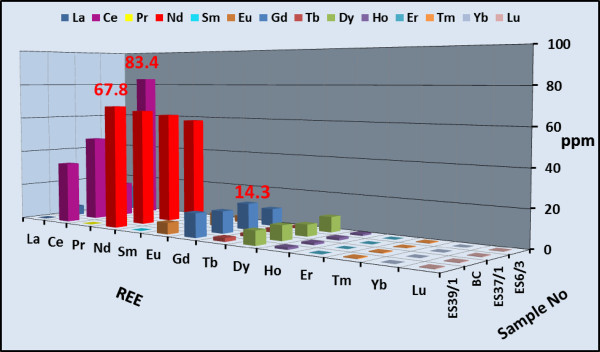
**Histogram shows the rare earth elements content in the altered basic dyke.**

The REE data of all altered microgranite and altered basic dykes samples are normalized against chonderite values. Tables 3 and 4) show the REE chonderite for the studied altered microgranite and altered basic dykes.Figures 16 and 17 show the normalized REEs patterns for the altered microgranite dyke and altered basic dyke. The REE plots show distinct differences exist in the nature and size of Eu anomaly. REEs pattern for the studied altered microgranite dyke samples shows distinct enrichment in LREE according to HREE with moderately to strongly negative Eu anomaly. REEs pattern for altered basic dyke samples shows strong enrichment in LREE according to HREE with slightly positive Eu anomalies.Figures 18 and 19 histograms show the highest REE values for altered microgranite and altered basic dykes. Lanthanum contents reach up to 50.19 ppm in altered microgranite dyke, while reach up to 14 ppm in altered basic dyke. Cerium contents reach up to 105.47 ppm in altered microgranite dyke, while reach up to 83.4 ppm in altered basic dyke. Neodymium contents reach up to 78.91 ppm in altered microgranite dyke, while reach up to 67.8 ppm in altered basic dyke.

## Conclusions

Multistage deformation, magmatic and hydrothermal processes accompanied by events of uranium mineralization with the associated rare metals and rare earths mineralizations in different episodes affected the biotite-muscovite granite of the El Sela shear zone. More than two distinct uranium-mineralizing events associated with altered microgranite and altered basic dykes are identified by petrographic study. Each event is marked by a deformational and alteration phase, which promoted fluid mobilization and deposition. This suggests that mobilization of fluids that formed uranium mineralization and the associated rare metals and rare earths mineralizations in El Sela shear zone are mainly tectonically-controlled. These hydrothermal fluids processes played an important role in concentrating the U, Zr, Nb, Y and REE in these dykes. Geochemical data indicating strong enrichment in some rare metals and rare earths content (Zr = 644, Y = 133, Nb = 136, Ga = 184, La = 60.6, Ce = 105.47, Nd = 78.91, Yb = 17.76 ppm) in these dykes. The chondrite normalised rare earth elements trends indicate strongly fractionated rare earth elements pattern with significant enriched of LREE according to HREE in both altered microgranite and altered basic dykes.
